# Combined iliac crest graft and short-scar pectoralis major flap for clavicular non-union reconstruction

**DOI:** 10.1080/23320885.2021.1962717

**Published:** 2021-08-11

**Authors:** Daniel Sattler, Hans-Philipp Springorum, Rafael Maria Armbruster, Maria von Kohout, Armin Kraus

**Affiliations:** a Division of Plastic and Aesthetic Surgery, Beta Klinik, Bonn, Germany; b Department of Plastic, Aesthetic and Hand Surgery, Otto-von-Guericke-University, Magdeburg, Germany

**Keywords:** Reconstructive surgery, head and neck, scar

## Abstract

Recently, we reported defect coverage in the clavicular region with a scar-sparing pectoralis major flap. We successfully combine this flap with a clavicular reconstruction by an iliac bone graft for non-union now. We propose this reconstructive combination for cases of clavicular non-union with lack of soft tissue coverage.

We report the case of a 23 year old male patient with clavicular non-union and infection with multiresistant gramnegative bacteria (4MRGN) after combat injury. Initial debridements and vacuum-assisted closure dressings had already been performed, leaving a soft-tissue defect (Figure 1) sized approximately 25 × 5 cm, with clavicular bone exposed over a length 20 cm and with an osseous defect of 2 cm. Reconstruction required both osseous reconstruction and soft tissue coverage. We combined a non-vascularized ilicac crest graft with a modified pectoralis major flap (PMF) described by us recently [1].

**Figure 1.**
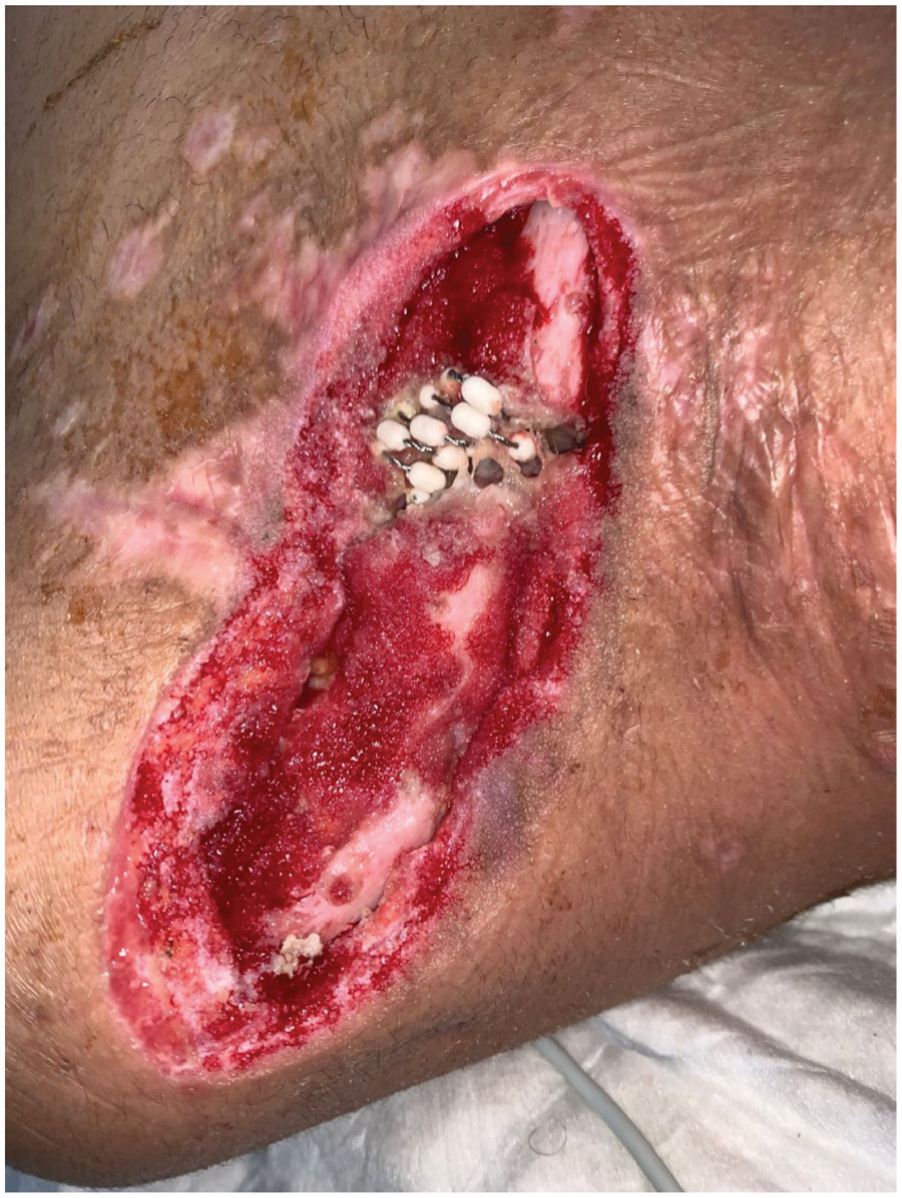
Soft tissue defect and clavicular non-union after repeated debridements and vacuum dressings. Bone defect was filled with antibiotic beads.

Clavicular non-union was resected over a length of 4 cm. An iliac crest graft of an adequate size was harvested, introduced into the defect and fixed with an osteosynthesis plate (Medartis locking plate, Figure 2). Subsequently, a PMF was elevated by a scar-sparing approach as described recently [1]. Briefly, the flap was elevated from an 8 cm long submammary fold incision, and dissected in cranial direction, using endoscopic light and electric cautery. The two vascularized pedicles were visually identified and caution was taken to avoid injury. The flap was pulled through a subcutaneous tunnel and used for coverage of the bone graft and the plate (Figure 3). A split-thickness skin graft was applied over the muscle flap. Post-surgical course was uneventful, with good graft embedding according to X-ray imaging 8 weeks after surgery (Figure 4) and good soft tissue healing (Figure 5). Postoperative range of motion for the shoulder 8 weeks after surgery was as follows: anteversion/retroversion 90°–0–40°; abduction/adduction 90°–0–40°; outer/inner rotation in shoulder neutral position 30°–0–45°; outer/inner rotation with elbow in 90° flexion 50°–0–50°.

**Figure 2.**
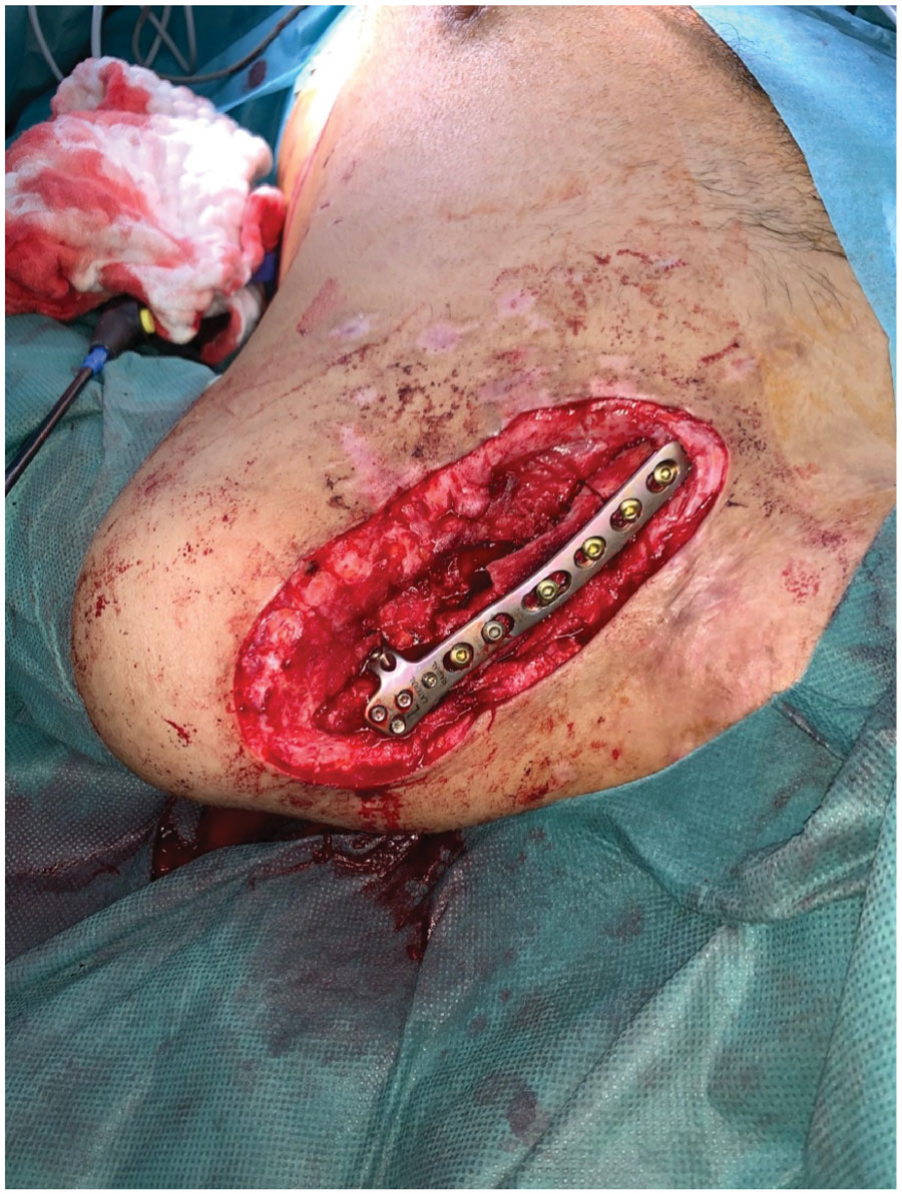
Interposition of a 4 cm iliac crest graft and fixation by a locking plate.

**Figure 3.**
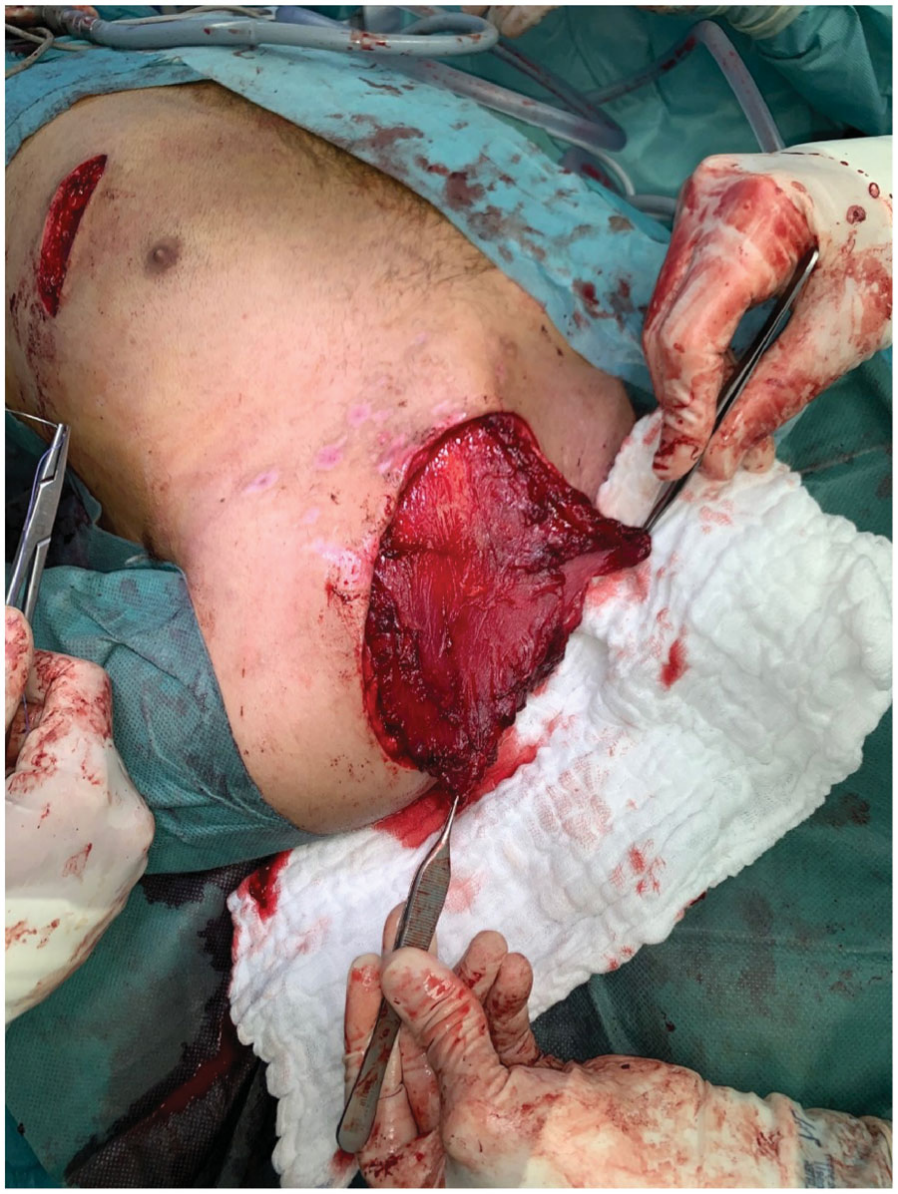
Coverage of the bone graft and the locking plate by a pectoralis major flap elevated via a short scar submammary incision.

**Figure 4.**
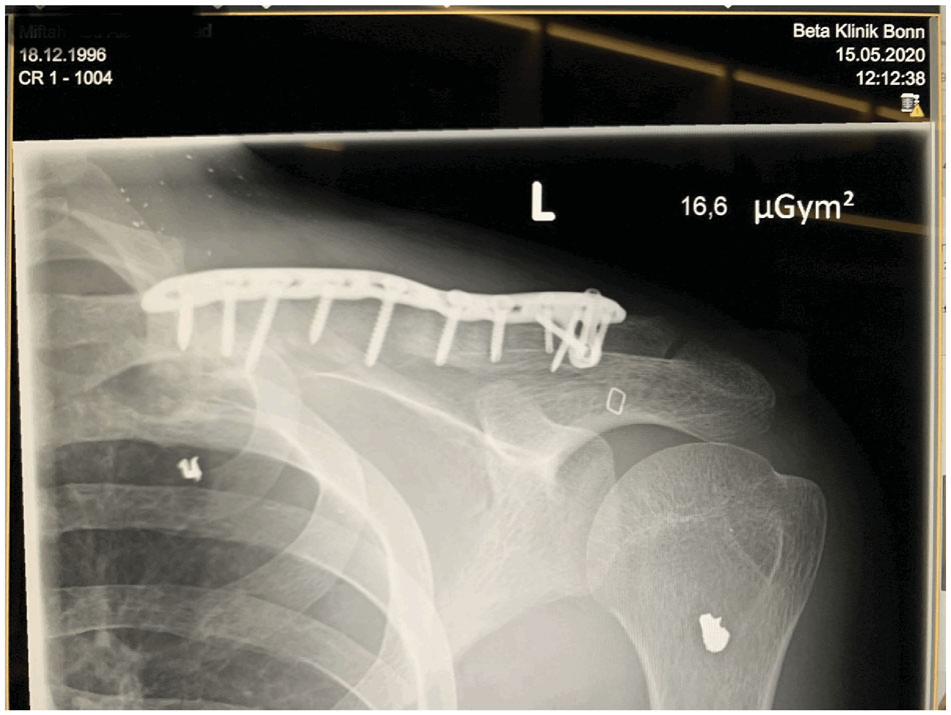
X-ray imaging 8 weeks after surgery shows good embedding of the bone graft and regular plate and screw positions.

**Figure 5.**
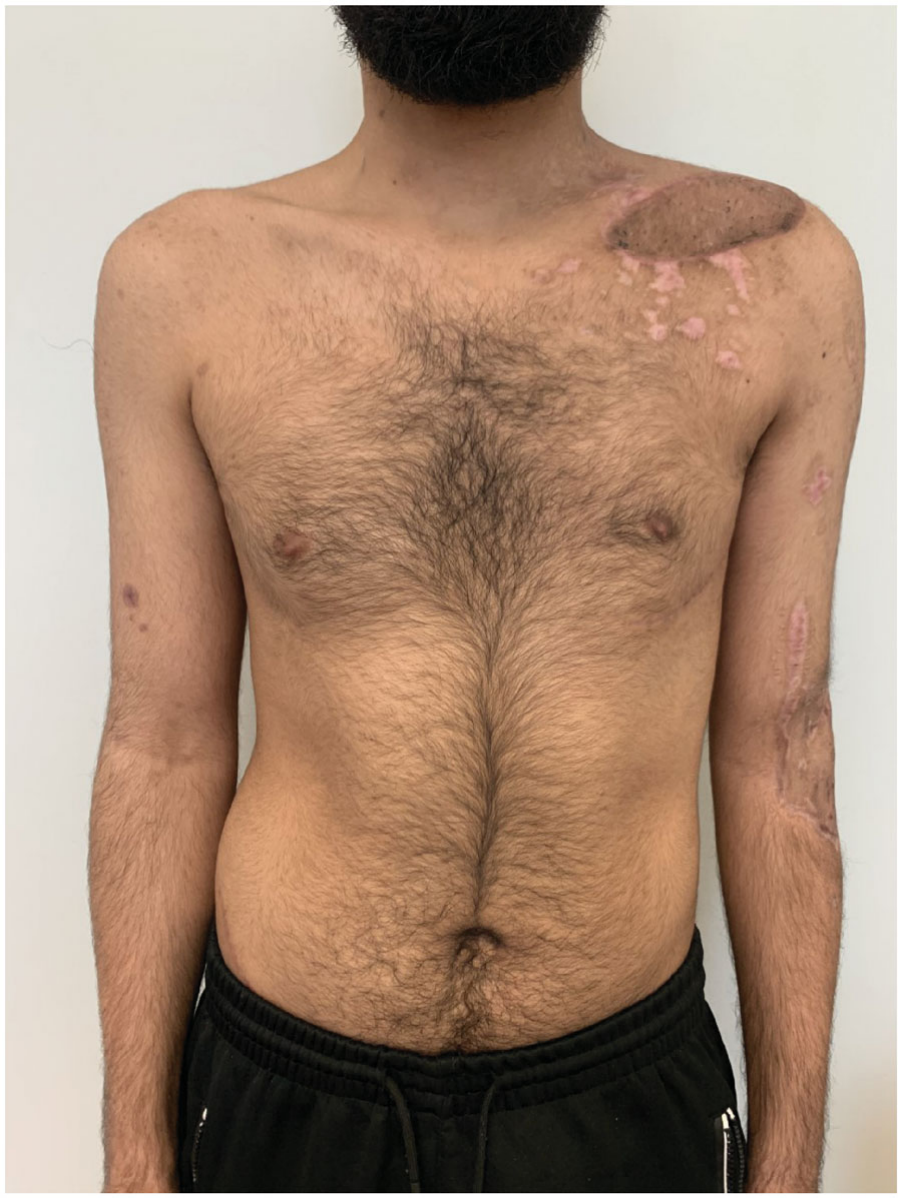
Postoperative result after 8 weeks: good defect coverage, inconspicuous scar and only minor impairment of the chest form can be observed.

## Discussion

In clavicular non-unions, healing rate can be improved by addition of a bone graft in addition to plate fixation alone [2]. Particularly when nonunion resection creates a bony defect, bone graft addition is required. It has been reported that a clavicular defect of over 1.5 cm benefits from bony bridging [3,4]. According to Wei and Mardini, bone defects up to 5 cm can be bridged by non-vascularized bone grafts [5]. In case of an additional soft tissue defect, as presented here, mechanical protection, vascularization and coverage by immunocompetent tissue seemed desirable to us, so that we chose a muscle flap. There is a choice for several free and pedicled muscle flaps, such as the deltoideus [6], the trapezius [7] and the latissimus dorsi [8], all with advantages and drawbacks. As alternatives, various free flaps could also be discussed. Decision is made, not least, by experience and comfort of the surgeon with the respective method.

We chose the short-scar PMF due to short operation time, facility, good vascularization and cosmesis. This combination with a nonvascularized iliac bone graft is proposed for situations with clavicular non-union and lack of soft tissue coverage.

## Conclusions

The short scar PMF in combination with iliac bone graft provides adequate soft tissue coverage, good bone healing, inconspicuous scar and good cosmesis for clavicular non-union reconstruction.
